# Cisplatin exposure causes c-Myc-dependent resistance to CDK4/6 inhibition in HPV-negative head and neck squamous cell carcinoma

**DOI:** 10.1038/s41419-019-2098-8

**Published:** 2019-11-14

**Authors:** Anthony M. Robinson, Richa Rathore, Nathan J. Redlich, Douglas R. Adkins, Todd VanArsdale, Brian A. Van Tine, Loren S. Michel

**Affiliations:** 1 Washington University in St. Louis School of Medicine, St. Louis, MO USA; 2 Medical College of Wisconsin, Milwaukee, WI USA; 3 Pfizer Oncology Research Unit, La Jolla, CA USA; 4 Memorial Sloan-Kettering Cancer Center, Monmouth, NJ USA

**Keywords:** Cancer therapeutic resistance, Oral cancer

## Abstract

The loss of p16 is a signature event in Human Papilloma Virus (HPV)-negative head and neck squamous cell carcinoma (HNSCC) that leads to increased Cyclin Dependent Kinase 4/6 (CDK) signaling. Palbociclib, a CDK4/6 inhibitor, is active for the treatment of a subset of HNSCC. In this study, we analyzed patient response data from a phase I clinical trial of palbociclib in HNSCC and observed an association between prior cisplatin exposure and CDK inhibitor resistance. We studied the effects of palbociclib on cisplatin-sensitive and -resistant HNSCC cell lines. We found that while palbociclib is highly effective against chemo-naive HNSCC cell lines and tumor xenografts, prior cisplatin exposure induces intrinsic resistance to palbociclib in vivo, a relationship that was not observed in vitro. Mechanistically, in the course of provoking a DNA damage-resistance phenotype, cisplatin exposure upregulates both c-Myc and cyclin E, and combination treatment with palbociclib and the c-Myc bromodomain inhibitor JQ1 exerts a synergistic anti-growth effect in cisplatin-resistant cells. These data show the benefit of exploiting the inherent resistance mechanisms of HNSCC to overcome cisplatin- and palbociclib resistance through the use of c-Myc inhibition.

## Introduction

Head and neck squamous cell carcinomas (HNSCC) are a collection of diseases, diagnosed in ~59,000 people per year, and responsible for ~12,000 deaths in the U.S. annually. The majority of HNSCC incidence (~40,000 cases) is attributed to tobacco exposure and smoking^1^. The molecular epidemiology of HNSCC is strongly determined by geographic location and anatomic subsite that dictates the genetics of these tumors. Among viral-related cancers, oropharynx cancers are increasingly caused by human papillomavirus (HPV)^2,3^. HPV-associated tumors usually lack mutations or deletions in cell cycle inhibitory proteins because the cell cycle machinery is disrupted by the E6 and E7 viral proteins. In contrast, tobacco-associated cancers acquire the capacity for unrestrained proliferation by a near ubiquitous loss of the tumor suppressor protein p16 (CDKN2A)^4^. p16 loss is tightly linked to smoking-related cancer and it serves as the biomarker for HPV-negative HNSCC^5,6^. In normal cells, p16 restrains the activity of the cyclin-dependent kinases 4 and 6 (CDK4/6). In HNSCC tumor cells, the loss of p16 confers CDK4/6 activity, resulting in hyperphosphorylation of the retinoblastoma protein (Rb)^7,8^. Thus far, there has been a distinct lack of therapies targeting the genetic alterations of HNSCC, with the epidermal growth factor receptor (EGFR) monoclonal antibody cetuximab being the only targeted agent to be approved^9^. Cisplatin chemotherapy remains the most effective first-line agent in recurrent and metastatic disease^10^. The epidemiologic and molecular data surrounding CDK4/6 and Rb in HNSCC suggest that CDK4/6 has promise as a therapeutic target in HNSCC.

Entry from G1 into S-phase is driven by the enzymatic activity of CDK4 and CDK6, which complex with one of the regulatory D-type cyclins (D1, D2, or D3)^11^. CDK4/6-cyclin D complexes promote hyperphosphorylation of Rb-family proteins (Rb1, RbL1/p107, and RbL2/p130), of which Rb1 is the best characterized^12^. Phosphorylation of Rb disables its capacity to function as a transcriptional repressor that sequesters the cell-cycle regulatory E2F transcription factor. These proteins are required to activate the S- and M-phase transcriptional programs needed for successful cell cycle progression. The importance of CDK4/6 and cyclin D1 in passing this checkpoint is highlighted by the observation that CDK4 and cyclin D1 are highly amplified in many tumors^13^. Moreover, CDK4 and cyclin D1 have been shown to be required for tumorigenesis in several experimental models^14–17^. CDK4/6 activity results in the activation of several genes, including cyclin E1 and cyclin E2^18^. Cyclin E is the regulatory subunit of CDK2, which further phosphorylates and completely inactivates Rb, leading to E2F release and cell cycle progression^19,20^. The functional relationship between the various CDK proteins is complex, and their biochemical roles have not been good predictors of their genetic function, as elucidated by mouse knockout studies^21^. Surprisingly, mice are able to survive inactivation of both CDK2 and CDK4 genes, and mammalian cell cycles with normal S-phase kinetics can be completed successfully in their absence^21,22^. These findings indicate the likelihood of significant functional redundancies in the cell cycle machinery, a probability which explains some of the difficulties observed with targeting cell cycle kinases.

Therapeutic targeting of the G1-S transition has been a longstanding goal of oncologic pharmaceutical development. Early CDK inhibitors, such as flavopiridol, were generally non-specific across multiple CDKs and exhibited limited activity in clinical trials^23,24^. Palbociclib (PD00332991) is unique as a selective inhibitor of CDK4/6, and is the first approved CDK inhibitor for the treatment of cancer^25^. Its original indication was for use in endocrine-resistant breast cancer. However, clear biomarkers of response to palbociclib treatment have yet to be identified, and neither amplification of CCND1 (coding for cyclin D1) or loss of p16 were definitively linked to response in breast cancer trials^26,27^. The lack of associated biomarkers that predict palbociclib response has fostered a great interest in the identification of mediators of therapy response and resistance. To date, pre-clinical models have offered some elucidation of potential determinants of palbociclib response; primarily, heightened CDK2 and cyclin E levels that have been observed in breast and pancreatic cancer models, have been shown to confer resistance to the drug^28,29^. Cyclin E is amplified in breast cancer and is overexpressed in pancreatic cancer^30^. Although cyclin E is not amplified or genetically altered in HNSCC, other inputs, such as EGFR, may elevate CDK2-cyclin E activity in this disease^4^.

An interesting feature of targeted therapy development (including immunotherapies) is that initial clinical testing usually takes place in the metastatic setting, in patients who have been exposed to cytotoxic chemotherapy. The utility of this strategy has some limitation, as it is well established that chemotherapy can alter the genetic landscape of residual tumors^31–33^. Whole-genome sequencing of leukemia cells has revealed that chemotherapy can result in the selection of aggressive clones that resist therapy^34^. Exposure to cisplatin can also alter the genome, resulting in a platinum-specific signature that includes an increase in a specific mutational profile (increased C > A mutations)^35^. Importantly, cisplatin is an essential component of both curative and palliative therapy for HNSCC. In this light, we investigated and now report the effects of cisplatin exposure on palbociclib sensitivity in both the pre-clinical and clinical settings in HNSCC. We demonstrate that cisplatin exposure confers intrinsic cross-resistance to the CDK4/6 inhibitor palbociclib. Using in vitro and in vivo analyses, we identify the underlying mechanism of this resistance, and demonstrate a clinically relevant mechanism for overcoming this resistance.

## Results

### Patients with cisplatin-resistant HNSCC show a reduced response to palbociclib treatment

**Fig. 1:**
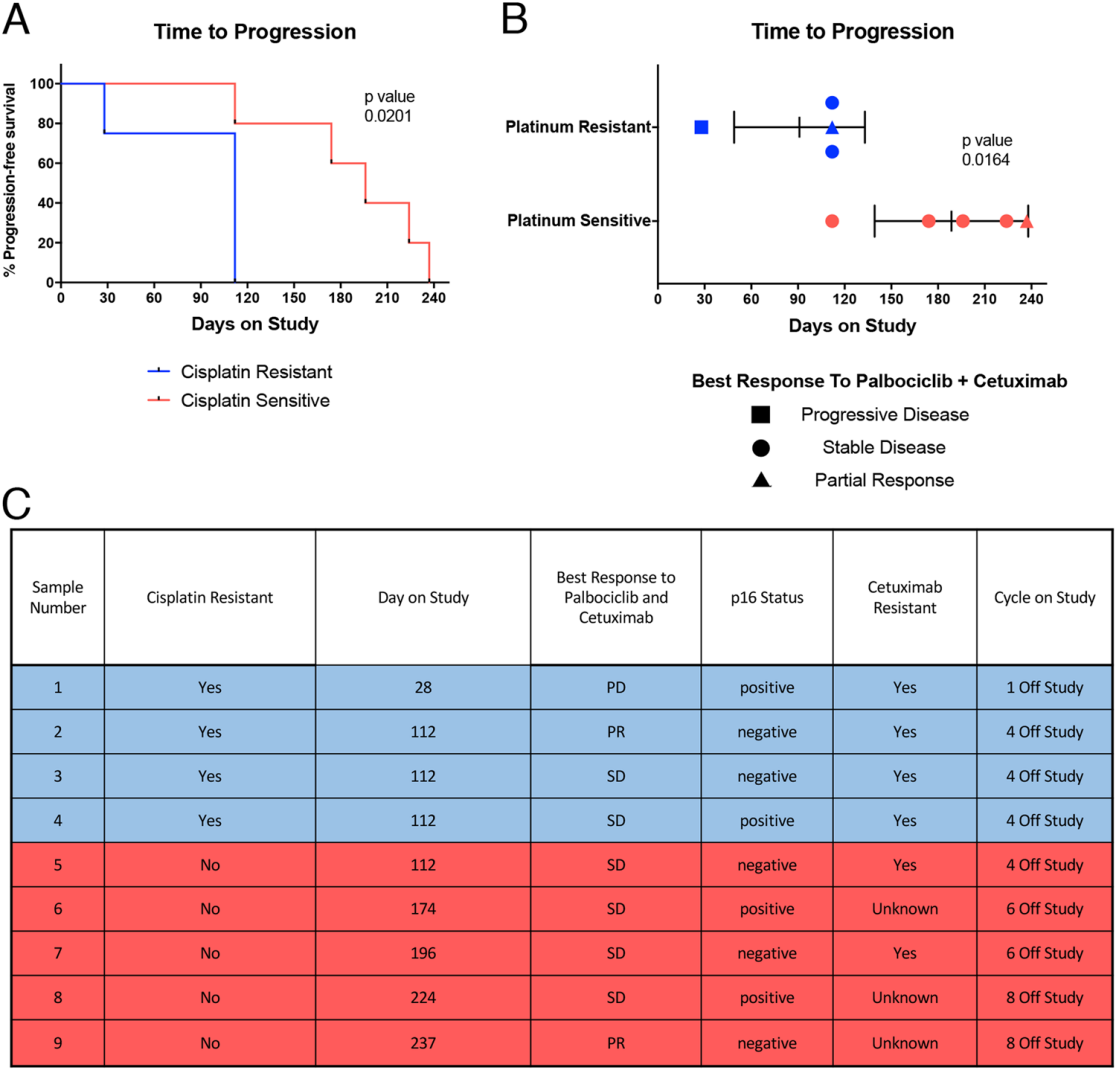
Patients with cisplatin-resistant HNSCC show a reduced response to palbociclib treatment. **a** Percent progression-free survival as shown by mean time to progression based on cisplatin resistance status in a Phase I clinical trial of palbociclib. Cisplatin-resistant patients (*n* = 4), cisplatin-sensitive patients (*n* = 5). Statistics stated are by Fisher’s exact test, error bars represent SD. **b** Mean time to progression highlighting disease response to palbociclib + cetuximab. **c** Patient characteristics for Phase I clinical trial (NCT02499120)

We previously reported the Phase I results of palbociclib combined with cetuximab in a cohort of either cetuximab- (6/9) or platinum- (4/9) refractory patients^36^. Palbociclib demonstrated activity in both platinum-sensitive and -resistant patients, but while the mean time to progression (TTP) in platinum-sensitive patients was 188.6 days, the mean time to progression in patients with platinum-resistant disease was shorter at 91 days (Fig. 1a) (*p* = 0.0201)^37^. As this data encompasses a patient set treated with a combination of cetuximab and palbociclib, this difference in TTP does not immediately distinguish the effects of palbociclib versus cetuximab in the pattern of response and resistance. However, cetuximab has activity (Fig. 1b) in cisplatin-resistant patients (patient characteristics presented in Fig. 1c)^38^, and therefore we considered that cisplatin exposure may be altering the efficacy of palbociclib specifically.

### Cisplatin resistance predicts palbociclib resistance in mice bearing HNSCC cell-line-derived xenografts

**Fig. 2:**
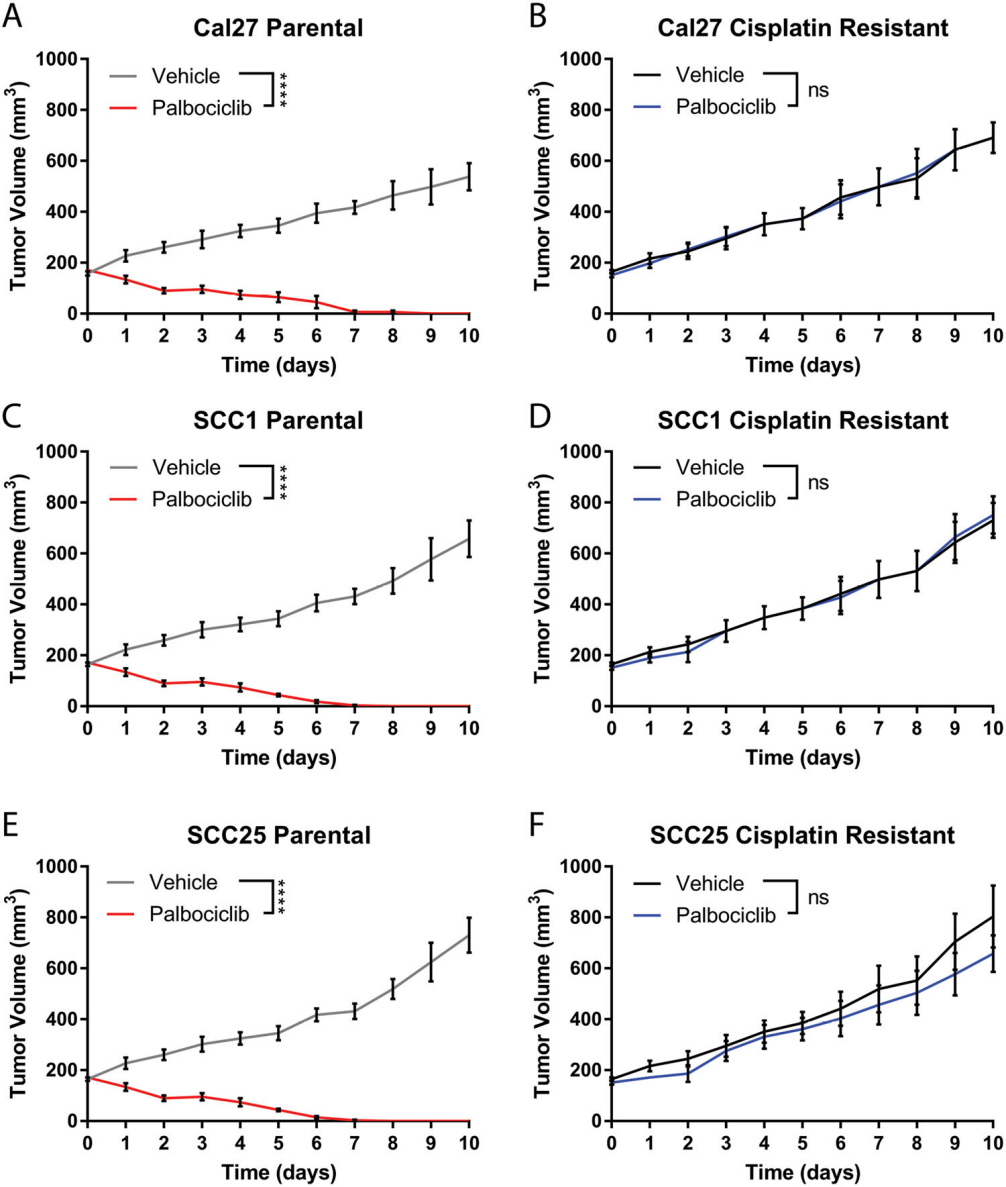
Cisplatin resistance predicts palbociclib resistance in mice bearing HNSCC cell-line-derived xenografts. Tumor growth curves of paired cisplatin-sensitive (**a**, **c**, **e**) and cisplatin-resistant (**b**, **d**, **f**) HNSCC cell lines, treated for 10 days with palbociclib (70 mg/kg, daily). *N* = 5 mice per group and measurements are summarized as mean ± SEM. (*p ≤ 0.05, **p ≤ 0.01, ***p ≤ 0.001, ****p ≤ 0.0001, by Student’s *t*-test)

To investigate the effects of cisplatin exposure on CDK4/6 inhibition in HNSCC, we identified palbociclib-sensitive HNSCC cell lines. We grew three commonly used HPV-negative, p16-negative cell lines (Cal27, SCC1, and SCC25) as xenografts in immunodeficient mice to ~200 mm^3^ in size and then treated the mice with palbociclib by oral gavage^39^. Tumor xenografts from each of the three cell lines responded significantly and nearly uniformly with evident tumor regression and complete loss of measurable tumor within ~10 days of treatment (*p* < 0.0001) (Fig. 2a, c, e). These data point to a dependency on CDK4/6 in these p16-negative HNSCC tumor lines.

Next, we determined the IC_50_ of cisplatin in vitro in these cell lines, and subsequently increased this value by serial exposure of cells to increasing levels of cisplatin using a previously published protocol^40^ (Supplemental Fig. 1A). Having established cisplatin-resistant cell lines, we sought to identify changes to the activity of palbociclib, but saw no consistent relationship between cisplatin exposure and palbociclib sensitivity in vitro, as seen by IC_50_ measurement (Supplemental Fig. 1B). We show that palbociclib treatment caused a significant increase in number of cells in G1, suggesting cell cycle arrest in both cisplatin-resistant cells and parental cells (Supplemental Fig. 2A–C). We also showed that with palbociclib treatment, there was a significant increase in cells accumulating in G2/S in cisplatin-resistant cells compared to parental lines, and a corresponding decrease in cells accumulating in G1 in cistplain-resistant cells compared to parental lines (Supplemental Fig. 2D–F). However, measuring DNA damage repair capacity in these cells by comet assay showed that cisplatin-exposed cells exhibited significantly diminished comet tails 1- and 4-h after radiation when compared to parental cells, indicating higher levels of DNA repair^41–43^ (Supplemental Fig. 3A, B). Interestingly, Cal27 cisplatin-resistant lines show increased comet tails compared to parental at baseline, suggesting decreased DNA repair in this line (Supplemental Fig. 3A, panel 1). This correlates with data showing that Cal27 has an overly active CDK4 pathway due to truncating mutations in SMAD4^44^, suggesting that the biological effects of cisplatin treatment, including increased DNA damage, could still be apparent in the resistant cell lines.

Targeted therapies may have differential effects in vitro as compared to in vivo^45^. Therefore, we tested the effects of cisplatin resistance in vivo by growing the parental and cisplatin-resistant (cisplatin-R) cells as xenografts in mice. Cisplatin-resistant cells were grafted into mice and allowed to form tumors ~200 mm^3^ in size, then treated with palbociclib. These tumors grew through treatment unabated at an equivalent rate to untreated tumors in all cell lines (Fig. 2b, d, f). These data indicate that cisplatin exposure causes resistance to palbociclib in palbociclib-naïve cells. Moreover, evidence of resistance is restricted to tumorigenic growth in vivo, and does not seem to extend to the two-dimensional tissue culture environment. To highlight the validity of this effect in vivo, we performed CD31 staining on sections of treated tumors, and found that palbociclib treatment caused a notable decrease in angiogenesis by CD31 when compared to vehicle control, implicating the microenvironment of the tumor as having a role in the efficacy of palbociclib (Supplemental Fig. 4A, B).

### Assessment of palbociclib pharmacodynamics in vivo shows significant CDK4 suppression

**Fig. 3:**
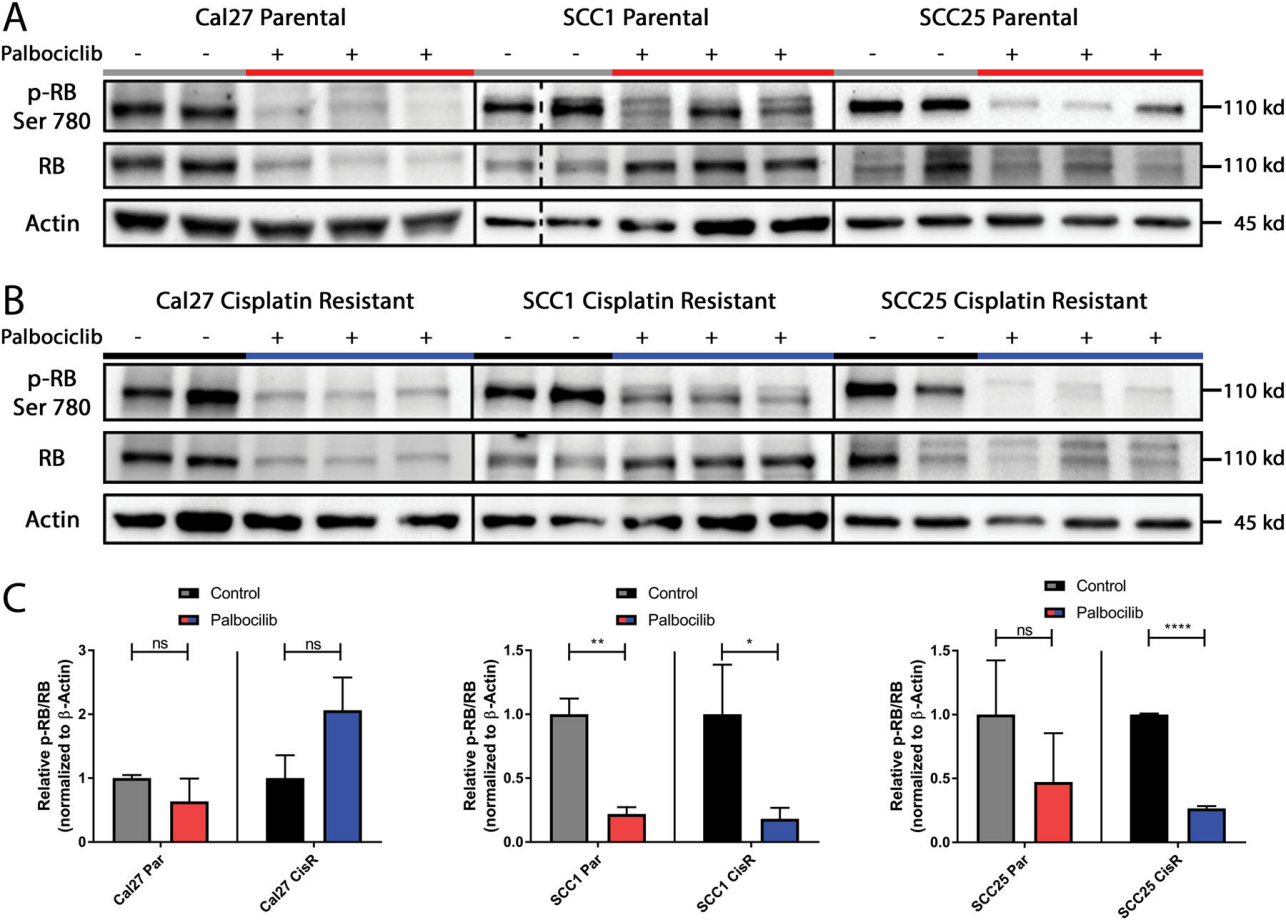
Assessment of palbociclib pharmacodynamics in vivo. **a** Representative immunoblots of Ser780 phospho-Rb, total Rb, and actin for tumor cell lysates obtained from xenografts of parental cell-line control (gray) and 48 h of palbociclib treatment (red). **b** Corresponding cisplatin-resistant cell-line control (black) and 48 h of palbociclib treatment (blue). **c** Quantification by photo densitometry was performed on bands using Image Lab Software, and the ratio of phospho-Rb/total Rb was calculated. All quantification is presented as mean ± SD. (*p ≤ 0.05, **p ≤ 0.01, ***p ≤ 0.001, ****p ≤ 0.0001, by Student’s *t*-test)

To investigate mechanisms of resistance to palbociclib, we identified the directed targeting and suppression of CDK4 by palbociclib. CDK4 phosphorylates Rb at serine 780, allowing for hyperphosphorylation of Rb. Tumor lysates were taken from control and cisplatin-resistant tumors, and levels of Rb phosphorylation at serine 780 were analyzed by immunoblot (Fig. 3a, b). We showed a decrease of phosphorylated Rb relative to total Rb in cisplatin-sensitive cells treated with palbociclib (Fig. 3c). In cisplatin-resistant cell lines, two of the three also showed a decrease in phosphorylated Rb relative to total protein (Fig. 3c), with CAL27 showing a lack of response due to SMAD4-related palbociclib resistance^44^. We therefore show that palbociclib treatment is decreasing phosphorylation of Rb, regardless of cisplatin-sensitivity status. Based on these results, we concluded that palbociclib adequately suppresses its target, CDK4.

### Cyclin E and CDK2 expression are enhanced in cisplatin-resistant samples and further elevated in cisplatin-resistant samples treated with palbociclib

**Fig. 4:**
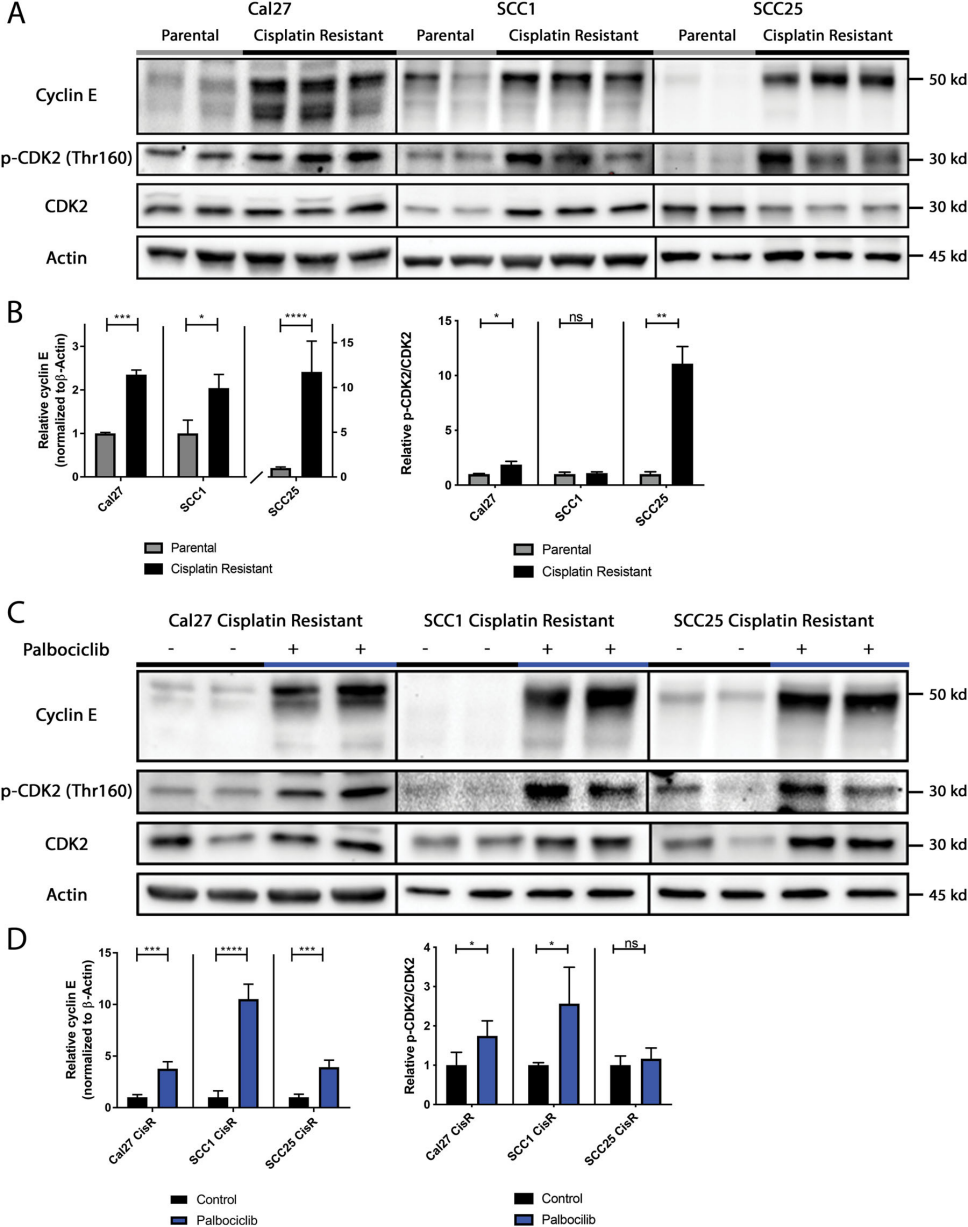
Cyclin E and CDK2 expression are enhanced in cisplatin-resistant samples and further elevated in cisplatin-resistant samples treated with palbociclib. **a** Representative immunoblots of cyclin E, phosphor-CDK2, total CDK2, and actin for tumor cell lysates obtained from untreated cisplatin naϊve (gray) and cisplatin-resistant xenografts (black). **b** Quantification by photo densitometry was performed on bands using Image Lab Software, cyclin E levels were normalized to β-actin loading control, and the ratio of p-CDK2/total CDK2 was calculated. **c** Representative immunoblots of cisplatin-resistant tumor cell lysates after 14 days of daily treatment with either vehicle control (black) or palbociclib (blue). Lysates were harvested 24 h after final dosing. **d** Accompanying quantification by photo densitometry. All quantification is presented as mean ± SD. (*p ≤ 0.05, **p ≤ 0.01, ***p ≤ 0.001, ****p ≤ 0.0001, by Student’s *t*-test)

To understand the phenomenon of unrestrained proliferation in palbociclib-treated tumors, we analyzed the levels of cell cycle regulatory proteins by immunoblot. Levels of cyclin A, D, and E were analyzed, as well as phosphorylated CDK2 and total CDK2 (Supplemental Fig. 5A, C, Fig. 4a), in tumor lysates that were either cisplatin-sensitive (parental) or cisplatin-resistant. Cyclin D has been most clearly linked to cisplatin resistance;^46^ however, we found no consistent pattern of alteration of cyclins A or D in cisplatin-resistant samples (Supplemental Fig. 5B, D). In contrast, these blots demonstrated that levels of cyclin E and phosphorylated CDK2 relative to total CDK2 increased in cisplatin-resistant tumors compared to parental lines (Fig. 4b).

To identify if palbociclib exposure in tumors might hyperactivate the cyclin E-CDK2 pathway, subsequently promoting palbociclib resistance in growing tumors, we looked at the above regulatory protein levels in tumors treated with palbociclib. Cisplatin-resistant tumors were treated daily for 2 weeks with either vehicle or palbociclib, and harvested 24 h after final dosing. We observed that cisplatin exposure caused increased cyclin E and CDK2 levels and activity (Fig. 4a, b), which has been previously linked to cisplatin sensitivity^47,48^, but also that palbociclib treatment further increased cyclin E and CDK2 activity (Fig. 4c, d). In addition, tumor growth rates for all lines treated with palbociclib or control were equivalent (Supplemental Fig. 5E).

### Increased c-Myc is a mediator of cisplatin- as well as palbociclib-resistance

**Fig. 5:**
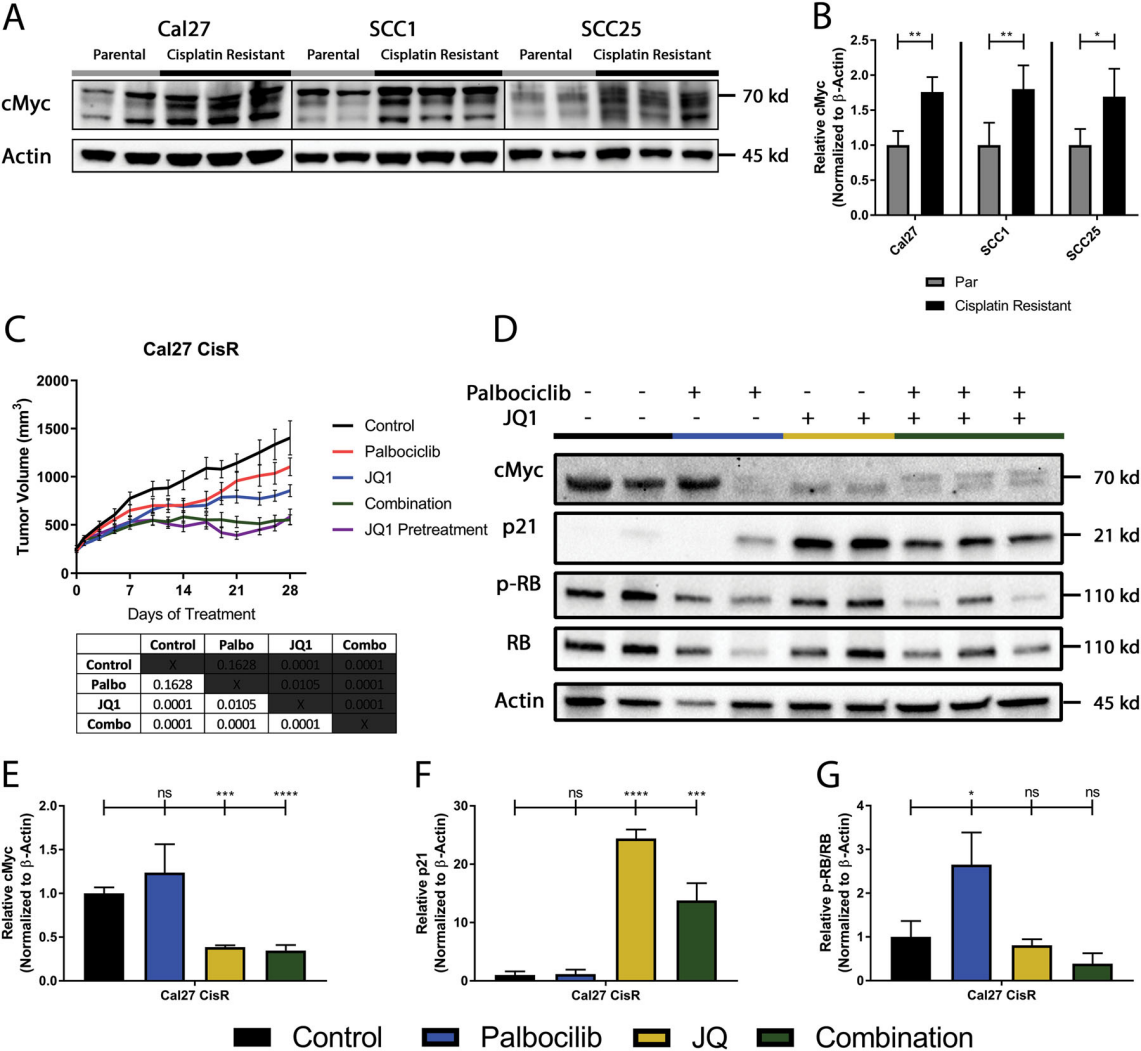
Cisplatin-resistant lines display up-regulated c-Myc expression and treatment with BET family protein inhibitor synergizes with Palbociclib treatment. **a** Representative immunoblots of c-Myc and actin for tumor cell lysates harvested from parental and cisplatin-resistant xenografts after 48 h of treatment with palbociclib. **b** Quantification of c-Myc and actin for tumor cell lysates harvested from parental (gray) and cisplatin-resistant (black) xenografts by photo densitometry was performed on bands using Image Lab Software, and normalized to β-actin loading control for c-Myc. **c** Tumor growth curves for cisplatin-resistant xenografts treated daily with vehicle (black), single agent palbociclib at 70 mg/kg (red), single agent JQ1 at 70 mg/kg (blue), combination therapy (green), or combination therapy pre-treated with JQ1 for the first 7 days with palbociclib added daily starting at day 7 (purple). *n* = 5 per group, summarized as mean ± SEM. **d** Representative immunoblots of c-Myc, p21, p-Rb, total Rb, and actin for tumor cell lysates harvested from xenografts treated with vehicle (black), palbociclib (blue), JQ1 (yellow), or combination therapy (green) after 28 days of treatment. **e** Quantification by photo densitometry normalized to β-actin loading control for c-Myc. **f** Quantification by photo densitometry normalized to β-actin loading control for p21. **g** Calculation by photo densitometry normalized to β-actin loading control of the ratio of p-Rb/total Rb. All quantification is presented as mean ± SD. (*p ≤ 0.05, **p ≤ 0.01, ***p ≤ 0.001, ****p ≤ 0.0001, by Student’s *t*-test)

c-Myc is an established mediator of cisplatin resistance^49–51^, and is also known to upregulate cyclin E^52,53^. Therefore, we compared the level of c-Myc in cisplatin-resistant versus parental tumors and found it to be significantly higher in the cisplatin-resistant lysates (Fig. 5a, b). To address whether this upregulation of c-Myc was an early or late event, we tested for c-Myc expression at early and late timepoints and found the induction to occur early in the process, by day 7 of cisplatin exposure (Supplemental Fig. 6). c-Myc is also linked to cell cycle progression by inhibiting the expression of p21, a protein that dephosphorylates and tags Rb for degradation. Based on this, we hypothesized that the bromodomain inhibitor JQ1, which is effective at inhibiting c-Myc^54,55^, might be effective in cisplatin- and palbociclib-resistant tumors. In vivo, neither palbociclib nor JQ1 as single agents were able to markedly overcome the resistance phenotype conferred by prior exposure to cisplatin, though JQ1 did exhibit a significant difference from vehicle control (Fig. 5c). Combination of JQ1 and palbociclib was sufficient to cause a significant reduction in tumor volume that began after 2 weeks of treatment (Fig. 5c). We hypothesized that increased c-Myc, either due to baseline levels in cisplatin-resistant cells or by palbociclib treatment, might be responsible for this delay in effect. Therefore, a subset of mice were pre-treated with JQ1 for 7 days before daily treatment with palbociclib commenced, in an attempt to reduce the delay. We found that JQ1 pre-treatment did not change the timing or efficacy of the combination, suggesting that the delay in efficacy was as a result of a natural adaptation to palbociclib treatment (Fig. 5c). Furthermore, we were able to show that this combination of JQ1 and palbociclib is synergistic in both parental and cisplatin-resistant cell lines (Supplemental Fig. 7). The only points corresponding with antagonism are associated with the lowest concentration of JQ1 that was used to treat SCC1 and SCC25 cisplatin-resistant cell lines (Supplemental Table 1).

To understand the underlying mechanism, we probed tumor lysates of single agent or combination treatment (Fig. 5d) and found that c-Myc expression was suppressed by JQ1 (Fig. 5e), and that treatment with JQ1 re-expressed p21 in this system (Fig. 5f). Single agent treatments had varying effects on Rb phosphorylation, with palbociclib increasing Rb phosporylation, while treatment with JQ1 resulted in modest a decrease to Rb phosphorylation. Importantly, in the combination of JQ1 and palbociclib, activation of Rb was more clearly achieved through the dual action of palbociclib- and p21-mediated (Fig. 5f) Rb dephosphorylation (Fig. 5g). These combination results provide rational for a strategy to mitigate the platinum-induced palbociclib resistance with the emerging class of BET bromodomain inhibitors.

## Discussion

HPV-negative head and neck squamous cell carcinoma (HNSCC) are a subset of tumors resulting from tobacco-related exposure or viral infection. Cisplatin is the most effective chemotherapy for the treatment of these cancers, but there is a high rate of clinical failure of cisplatin due to inherent or acquired resistance^56^. Unexpectedly, Phase 1 clinical trial data utilizing palbociclib identified that palbociclib has decreased activity in cisplatin-resistant HNSCC, suggesting a signaling pathway linking the two therapies and driving potential resistance to treatment. Understanding the biological shifts that these first-line therapies enact in cancer will allow for the development, sequencing, and greater utilization of targeted therapies.

**Fig. 6:**
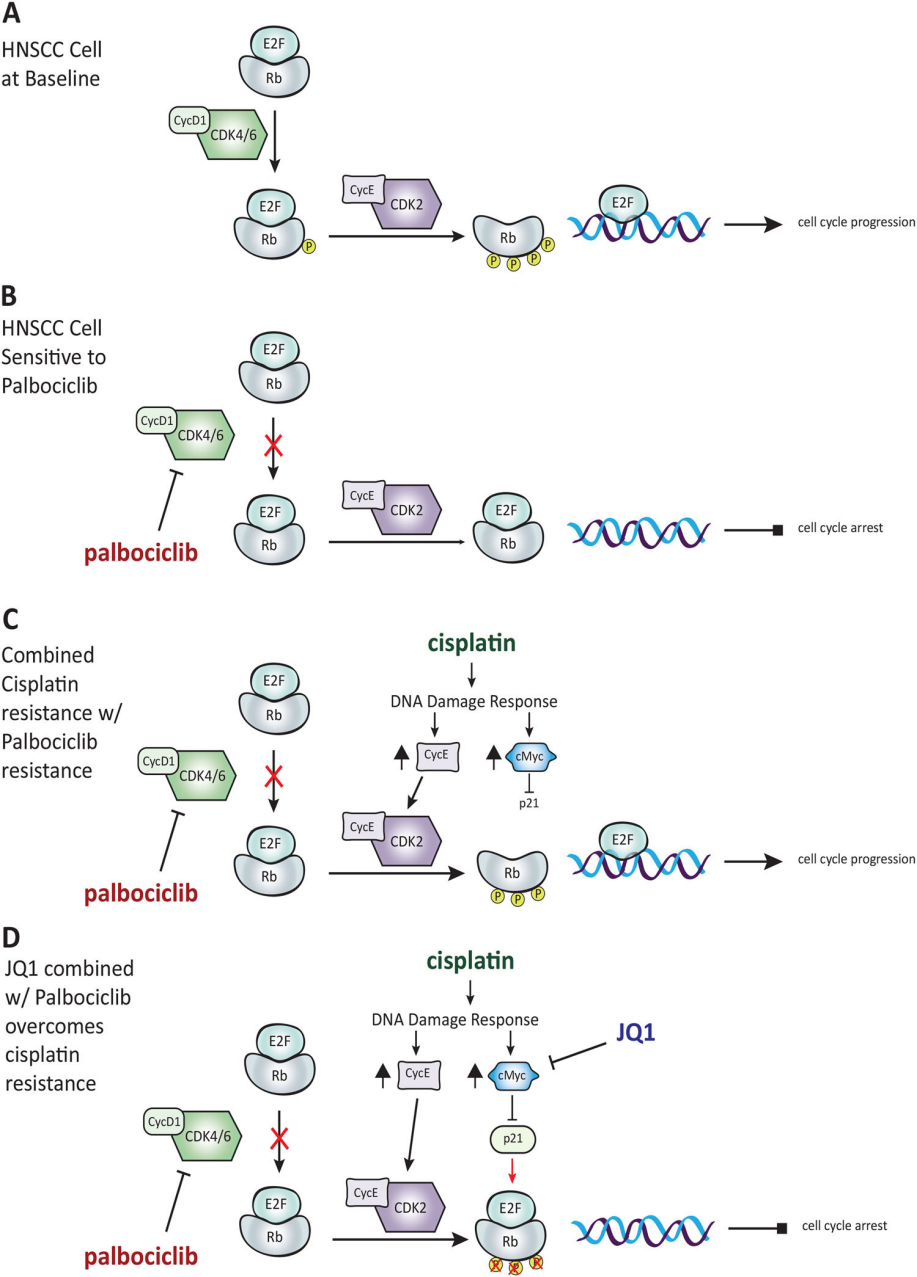
Proposed model of JQ1 and palbociclib activity in cisplatin-sensitive and resistant cell lines. **a** HNSCC cell at baseline. **b** HNSCC cell sensitive to cisplatin. **c** Cisplatin resistance confers palbociclib resistance. **d** JQ1 combined with palbociclib overcomes cisplatin resistance

HNSCC is characterized by loss of p16, a tumor suppressor protein that restrains the activity of cyclin-dependent kinases 4/6 (CDK4/6), and allows for the hyperphosphorylation of Rb. In normal tissue, the active state of Rb sequesters E2F transcription factors, thereby preventing cell cycle progression. Rb becomes inactivated through a stepwise process of phosphorylation by cyclin-dependent kinases; initial phosphorylation occurs through the complex of CDK4/6-cyclin D1, with further phosphorylation occurring by the complex of CDK2-cyclin E. Following hyperphosphorylation, Rb releases E2F, which then acts as a transcription factor to drive cell cycle progression to S-phase (Fig. 6a). Palbociclib interrupts this process at the first step, inhibiting the activity of CDK4/6/cyclin D1 and blocking further phosphorylation of Rb (Fig. 6b). Importantly, the CAL27 cell-line has been shown to have a truncating mutation in SMAD4^44^, which can cause aberrant activation of the CDK4 pathway^57^, downregulation of the TGF-β pathway, and subsequent resistance to palbociclib treatment^58^ and other CDK4 inhibitors^59^. This may explain the limited efficacy of palbociclib in CAL27, and further emphasizes the importance of identifying therapies that make palbociclib treatment more effective.

Cyclin E has been shown to overcome the anti-proliferative activity of palbociclib in Rb positive cells in vitro, as its cognate kinase CDK2 can inactivate Rb and promote cell cycle progression^29,60^. We observed cyclin E overexpression and hyperactivation of CDK2 in the cisplatin-resistant lines. Recent studies have implicated CDK2 as a potential mediator of DNA damage resistance^61,62^. Therefore, upregulation of cyclin E and p-CDK2 in cisplatin-resistant cells may contribute to the DNA damage response phenotype observed in these cells. Therefore, the upregulation of cyclin E after cisplatin exposure was an unexpected finding in our model system. However, our data is consistent with reports that cisplatin exposure can activate CDK2, promoting the phosphorylation of Rb and the subsequent release of E2F for continued cell cycle progression^63^ (Fig. 6c).

The ability of oncogenes and tumor suppressor genes to mediate resistance to cytotoxic chemotherapy is well established. Early experiments with p53 loss of function revealed a clear effect on chemoresistance^64,65^. Subsequently, c-Myc was found to be a driver of cisplatin-specific resistance^49–51^. Our data was consistent with these observations, showing that c-Myc is significantly upregulated in cisplatin-exposed cells. To this end, we utilized the BET bromodomain inhibitor JQ1 to inhibit c-Myc, in conjunction with palbociclib treatment. The inhibition of c-Myc allows for the re-expression of p21, driving the dephosphorylation of Rb. This overcomes the activity of CDK2-cyclin E and augments the efficacy of palbociclib (Fig. 6d). We were able to identify synergy between palbociclib and JQ1, showing that the combination of these therapies can overcome cisplatin resistance and be more effective than either agent alone. This provides a pre-clinical rationale for future clinical trial development^66^. It is likely that strategy using dual BET bromodomain with a CDK4/6 inhibitory strategy may be useful in treating a significant percentage of patients with cisplatin-resistant HNSCC.

Importantly, the relevance of these findings can be attributed to other cisplatin-resistant cancers. Bromodomain inhibitors such as JQ1 have been shown to down-regulate MYC expression in MYC-amplified tumors, which sensitizes ovarian cancer cells to platinum-based therapy^67^. In addition, the combination of JQ1 with cisplatin in ovarian cancer cells has shown increased survival and decreased tumor outgrowth^68,69^. These findings highlight the importance of identifying a synergistic treatment that can overcome the resistance phenotype in HNSCC and other platinum-resistant cancers.

This data suggests that further exploration into the mechanism by which c-Myc is upregulated will be necessary in order to fully understand the role of p21 and Rb in the response to palbociclib and JQ1. If modifications to SMAD4 and other factors in the CDK4 pathway have a role to play in the upregulation of c-Myc, this could stratify patients that could benefit from combination therapy. Noting the effect that prior EGFR treatment has within this system will also be useful to study, as this data was based on a clinical trial where most of the patients were exposed to cetuximab. Additionally, we show that palbociclib treatment reduces levels of total Rb in parental and cisplatin-resistant cell lines. This is an important area for future study, as the mechanism of total Rb reduction may be a consequence of G1 arrest, as previously reported^70^, further implicating palbociclib as a cell cycle arrest agent. Finally, CD31 staining showed a marked decrease in angiogenesis in palbociclib-treated tumors compared to vehicle control (Supplemental Fig. 4). This partially explains the discordance in our in vitro versus in vivo models. Future work exploring the role of the microenvironment in response to cisplatin resistance could yield new avenues for therapeutic exploration.

A major limitation of this research is the cisplatin-resistant cell lines that were utilized. The cell lines were created by increasing concentrations of cisplatin treatment, rather than deriving cell lines from cisplatin-resistant patients or creating a resistance model in vivo. Furthermore, the microenvironment may play a strong role in tumor response to combination treatment, suggesting that there may be differences in the biology of cisplatin-resistant tumors that develop resistance in vivo, in the presence of microenvironmental factors.

## Materials and methods

### Cell line maintenance

UM-SCC1 (referred to as SCC1, provided by the University of Michigan), SCC25 (ATCC), and Cal27 (ATCC) cell lines were cultured at 37 °C in 5% CO_2_, in Dulbecco’s Modified Eagle Medium/F12 Ham’s Nutrient Mix (DMEM/F12) (Gibco #11330–032) supplemented with 10% FBS (Gibco #16140-071), Penicillin-Streptomycin 100 × (10,000 U/mL) (Life Technologies, Grand Island, NY 15140122), and Hydrocortisone (400 ng/mL) (Sigma H-0888). Cells were regularly passaged with 0.05% Trypsin/EDTA (500 mg/ml Trypsin, 200 mg/ml EDTA) (Life Technologies, Grand Island, NY). All lines were regularly tested for mycoplasma.

### Cisplatin-resistant line generation

Cell lines were exposed to one quarter of the initial IC50 concentration of cisplatin for 1 week, then allowed to recover until normal growth resumed. Cycles of increasing drug concentration were applied to cell lines until confirmed resistance by Alamar blue assay.

### Xenograft studies

Mouse xenograft experimental protocols were approved by the Institutional Animal Care and Use Committee (IACUC) at Washington University in St. Louis. Animals were maintained and evaluated under pathogen-free conditions following IACUC guidelines (St. Louis, MO). Athymic nude mice (4–6-week-old females) were obtained from Jackson Laboratories (Bar Harbor, ME). A suspension of 2.0 × 10^6^ cells was injected subcutaneously into both flanks of each mouse. Length and width of tumors were measured several times per week, and volumes were calculated using the formula (length x width^2^)/2. Tumors were allowed to reach an average volume of 200 mm^3^ before being randomly separated into treatment groups. Mice were treated with vehicle control, palbociclib in 0.05 N lactic acid by oral gavage daily at 70 mg/kg body weight, or (+) JQ1 in 5% DMSO, 10% 2-Hydroxypropyl-β-cyclodextrin by intraperitoneal injection at 70 mg/kg body weight daily. Five mice per treatment group were utilized for all animal studies, with no pre-determined size estimate. Investigators were not blinded to mouse treatment conditions at time of measurement. Mice were killed according to IACUC approved protocol upon reaching two centimeters diameter in one dimension, or 1 day after last treatment. Tumor material was harvested for (1) protein analyses by immunoblot after snap freezing in liquid nitrogen, and (2) histological studies by formalin fixation. Tumor growth curves are summarized as mean ± SEM, with similar variance between all groups being compared. (*p ≤ 0.05, **p ≤ 0.01, ***p ≤ 0.001, ****p ≤ 0.0001, by Student’s *t*-test).

### Immunoblot analysis

For xenografted tumor protein expression, three tumor portions per treatment group were homogenized using pestles and lysed in RIPA buffer for 20 min before sonication, followed by centrifugation for 10 min at 4 °C, and supernatant collection. Total protein concentrations were determined by Quick Start™ Bradford Assay (BioRad). In all, 40 µg of protein lysate were added per sample, diluted in millipure H_2_O to 20 µL, and diluted further with 5x SDS-PAGE Loading Buffer. Samples were boiled at 95 °C for 5 min and ran through an SDS-PAGE gel. Samples were electrotransferred onto 0.2 µm PVDF membranes. Membranes were blocked with 5% milk in PBS-0.1% Tween20 for 30 min at room temperature. Primary antibodies diluted in 3% BSA, 0.02% NaN_3_ solution were incubated overnight on a rocker at 4 °C. After primary antibody incubation, membranes were washed three times in 1x PBS-0.1% Tween20 for 8 min each. Species-specific HRP conjugated secondary monoclonal antibodies were diluted in 5% milk in 1 × 0.1% Tween20 at a concentration of 1:5000 and incubated for 1 h at room temperature. After secondary antibody incubation, membranes were washed three times in 1 × 0.1% Tween20 for 8 min each. SuperSignal West Femto or Dura Maximum Sensitivity Substrate (Thermo) was used for visualization. Unused lysates were stored at −80 °C. Images were acquired using Chemidoc XRS + imaging system and Image Lab Software. The antibodies as listed: Cell Signaling: anti-p-Rb #9307, anti-Cyclin E (he12) #4129, anti-p-CDK2 #2561, anti-Cyclin D1 #2978, anti-p21 #2947. R&D: anti-CDK2 AF4654, anti-Cyclin A2 AF5999. Abcam: anti-c-Myc ab32072, anti-Rb ab85607. Sigma: anti-β-Actin A1978. Quantification of western blots are summarized as mean ± SD, with similar variance between all groups being compared. (*p ≤ 0.05, **p ≤ 0.01, ***p ≤ 0.001, ****p ≤ 0.0001, by Student’s *t*-test).

### Cell cycle analysis

For cell cycle analysis, 1 × 10^6^ cells were treated with palbociclib for 24 h. Media was removed and cells were trypsinized and collected for analysis. PI staining solution was made using 0.1% sodium citrate, 0.03% NP40, 0.02 mg/mL RNase A, and 25 μg/mL propidium iodide. Cells were resuspended in 350 μL of PI staining solution (for a final concentration of 1 mg/mL PI) and incubated for 20 min at 4 °C. Staining was analyzed by fluorescence activated cell sorting (FACS) using a BD FACScan flow cytometer. In total, 10,000 events were captured and analyzed using the cell cycle analysis program in FlowJo (version 10) analysis software (Becton, Dickinson, and Company). This analysis was conducted in triplicate with biological replicates. Quantification of analysis is summarized as mean ± SD, with similar variance between all groups being compared. (*p ≤ 0.05, **p ≤ 0.01, ***p ≤ 0.001, ****p ≤ 0.0001, by Student’s *t*-test).

### JQ1 and palbociclib cell death and synergy studies in vitro

Cells were plated at 4–6 × 10^3^ cells per well in 96-well plates, depending on cell line. The following day, media was changed to media supplemented with DMSO, Palbociclib, JQ1, or combination. Doses were dependent on cell line. For cell death for IC50, Alamar blue diluted 1:10 in media was added, and plates were incubated 2–3 h before analysis by spectrophotometry. For synergy, plates were loaded into IncuCycte Zoom platform and assayed for 72 h. Proliferation data was determined by percent confluence readout and converted to percent growth inhibition compared to vehicle control, and cell death was measured by YOYO-1 Iodide. All assays were conducted in triplicate with biological replicates. CalcuSyn software (Cambridge, UK) was utilized to measure combination index and Graphpad Prism 8 software was utilized for statistical analysis. Quantification of analysis is summarized as mean ± SD, with similar variance between all groups being compared. (*p ≤ 0.05, **p ≤ 0.01, ***p ≤ 0.001, ****p ≤ 0.0001, by Student’s *t*-test).

### DNA damage comet assay

Cells were plated at 2 × 10^5^ cells per well in 6-well plates. The following day, one hour after treatment conditions, cells were trypsinized and centrifuged in 15 mL tubes prior to irradiation. Cells were treated with either 10 gray or 30 gray ionizing radiation depending on line, using the Small Animal Radiation Research Platform (SARRP) by xStrahl. Cells were then assayed using Trevigen Comet Assay (4252-50-K) according to the Trevigen protocol for alkaline conditions. DNA was stained using SYBR-Green fluorescent dye and visualized on an Olympus IX70 microscope with an Olympus DP72 camera using a ×20 objective lens. CellSens Entry software was used for capturing images. OpenComet plugin from ImageJ was used to analyze the resulting comets. All assays were conducted in triplicate with biological replicates, and 40 images were collected per run. Quantification of analysis is summarized as mean ± SD, with similar variance between all groups being compared. (*p ≤ 0.05, **p ≤ 0.01, ***p ≤ 0.001, ****p ≤ 0.0001, by Student’s *t*-test).

## Supplementary information

Supplemental Figure 1

Supplemental Figure 2

Supplemental Figure 3

Supplemental Figure 4

Supplemental Figure 5

Supplemental Figure 6

Supplemental Figure 7

Supplemental Table 1

Supplemental Legends
