# Supplementary figures and images for: Cisplatin exposure causes c-Myc-dependent resistance to CDK4/6 inhibition in HPV-negative head and neck squamous cell carcinoma
Source: Cell Death Dis. 2019 Nov 14;10(11):867. doi: 10.1038/s41419-019-2098-8 (PMC6856201; doi:10.1038/s41419-019-2098-8)

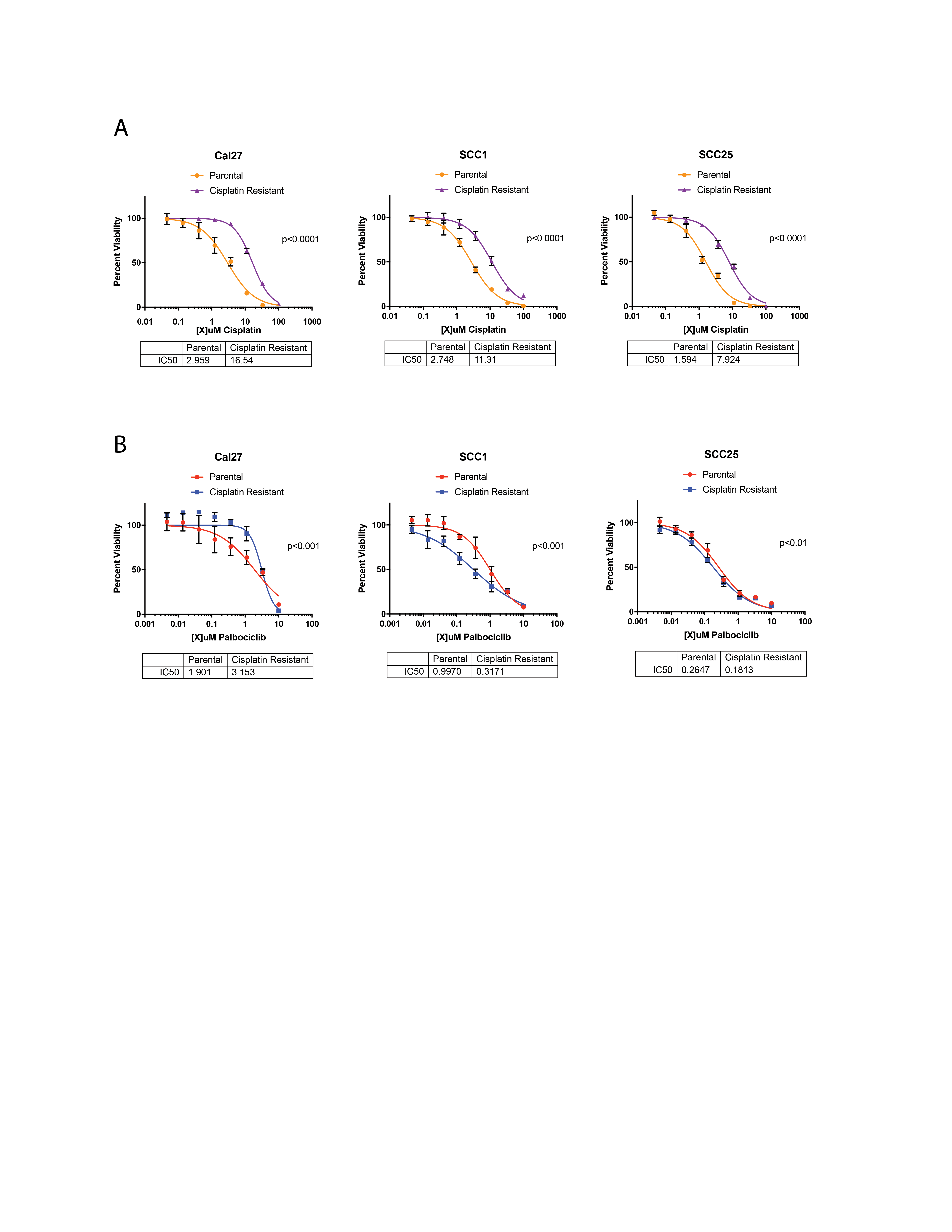

Supplement: Supplementary file 1 — Supplemental Figure 1 [file 41419_2019_2098_MOESM1_ESM.png]

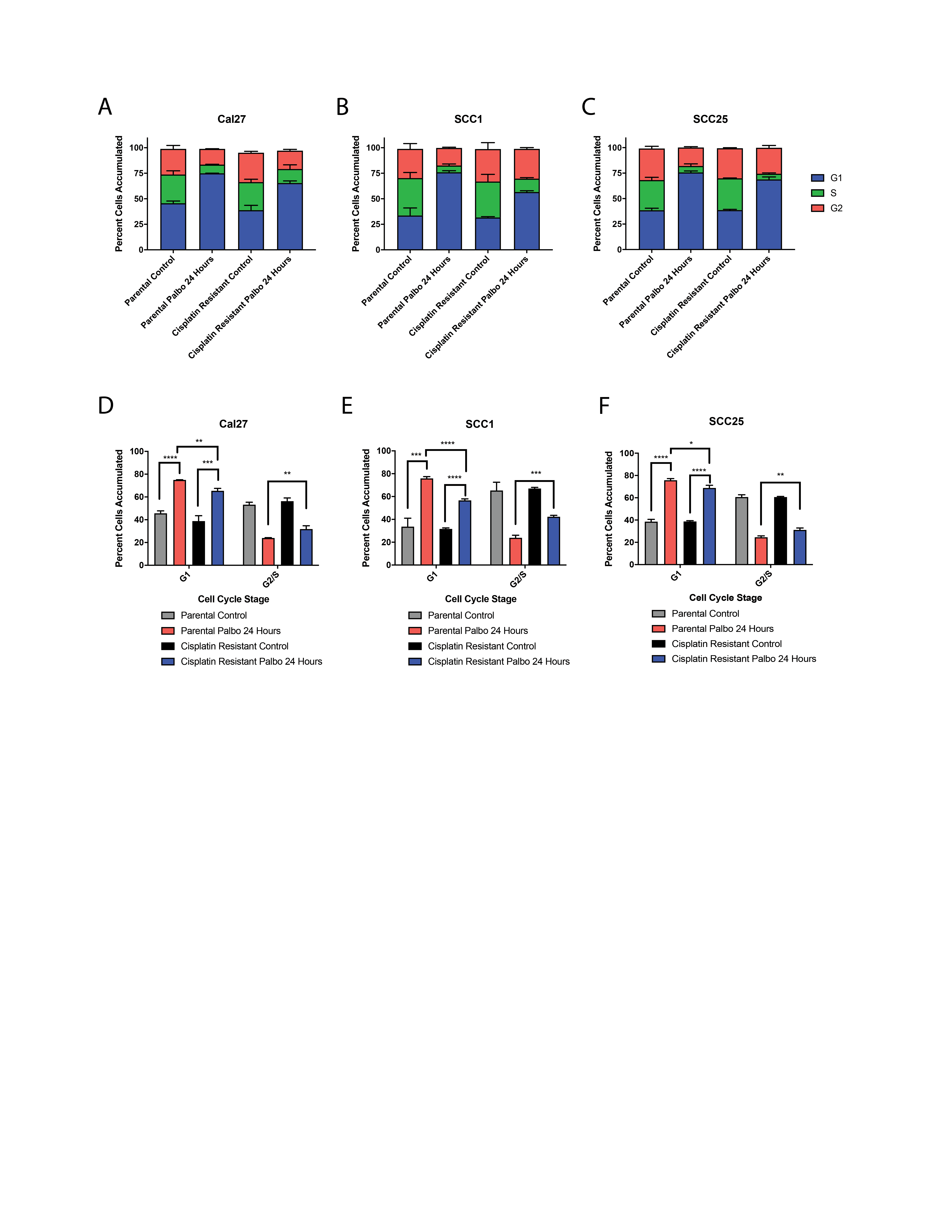

Supplement: Supplementary file 2 — Supplemental Figure 2 [file 41419_2019_2098_MOESM2_ESM.png]

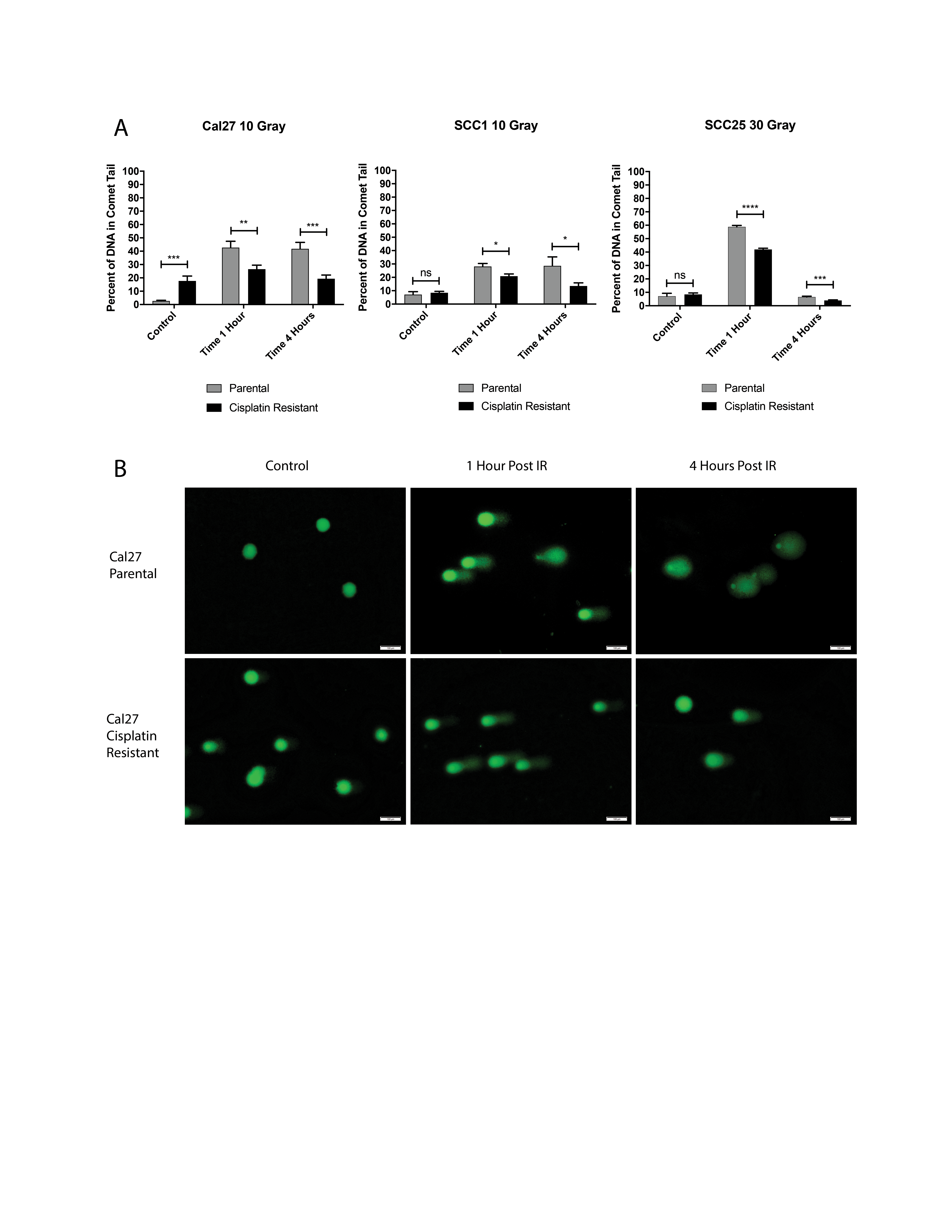

Supplement: Supplementary file 3 — Supplemental Figure 3 [file 41419_2019_2098_MOESM3_ESM.png]

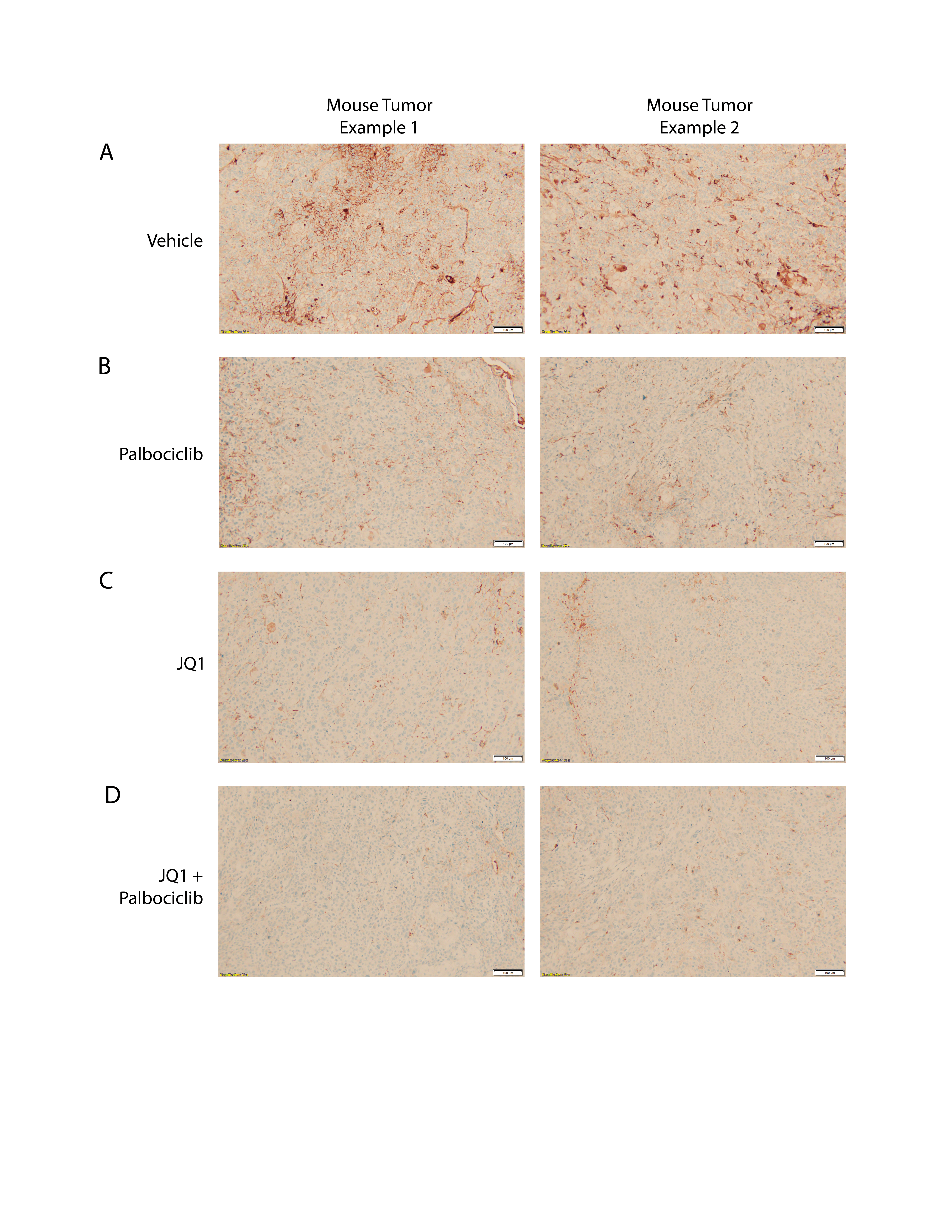

Supplement: Supplementary file 4 — Supplemental Figure 4 [file 41419_2019_2098_MOESM4_ESM.png]

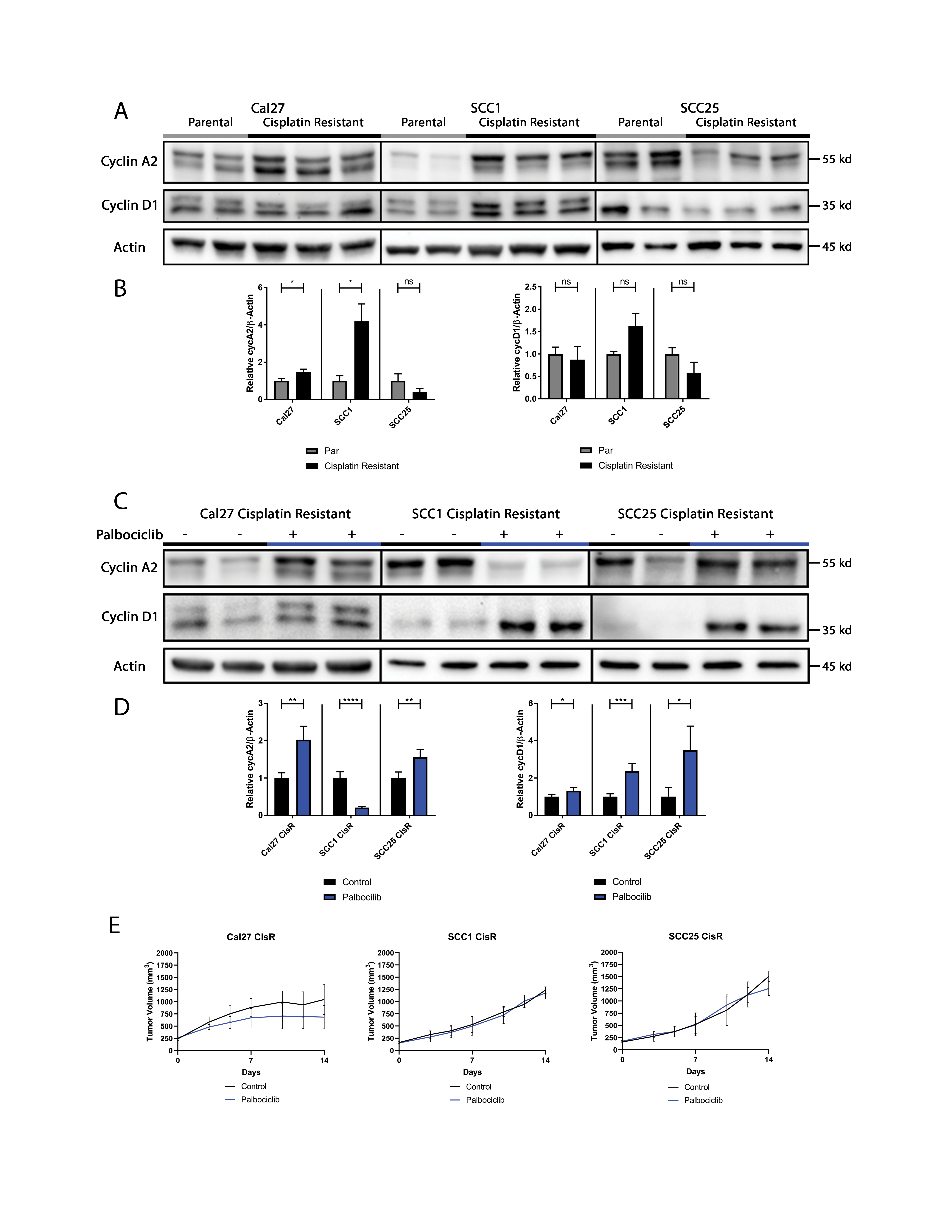

Supplement: Supplementary file 5 — Supplemental Figure 5 [file 41419_2019_2098_MOESM5_ESM.png]

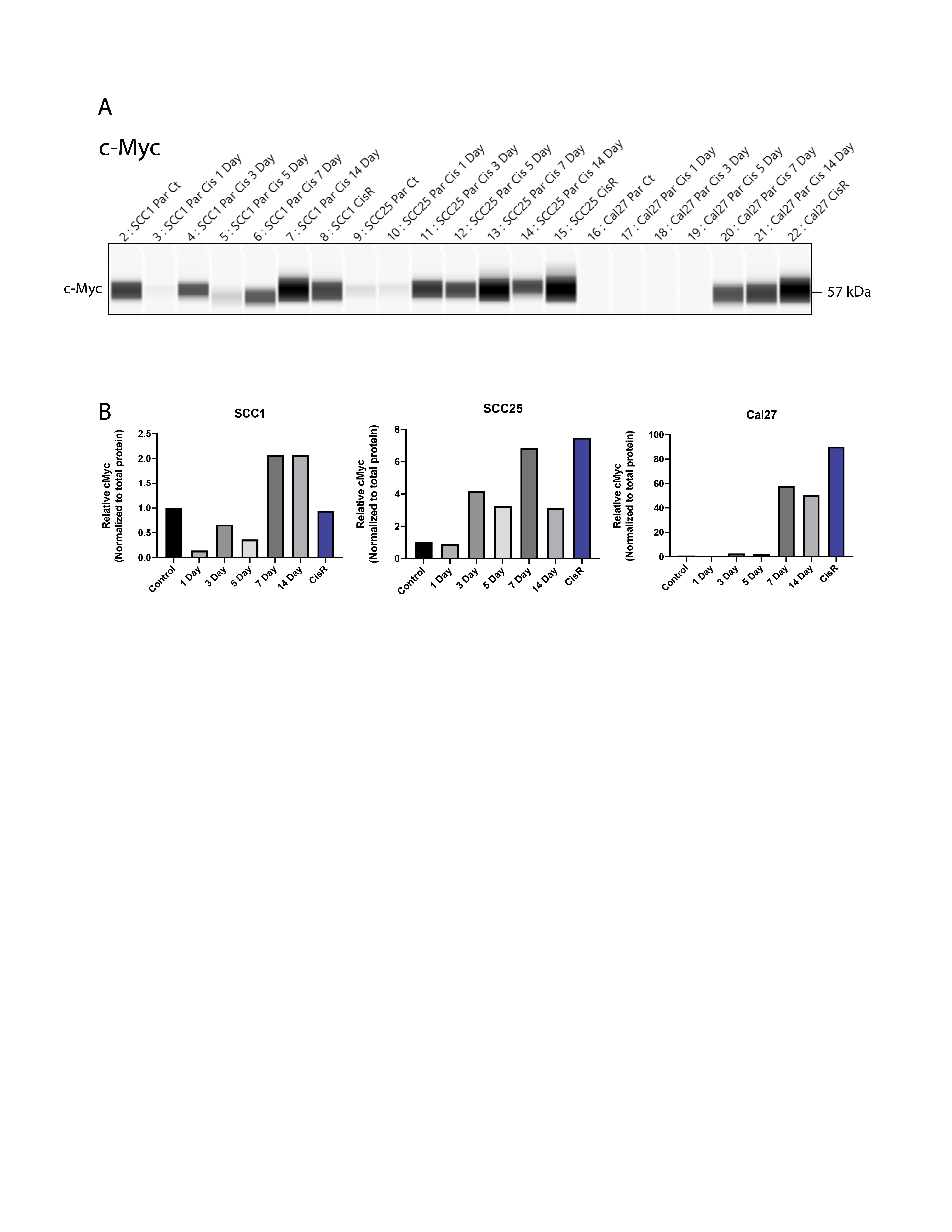

Supplement: Supplementary file 6 — Supplemental Figure 6 [file 41419_2019_2098_MOESM6_ESM.png]

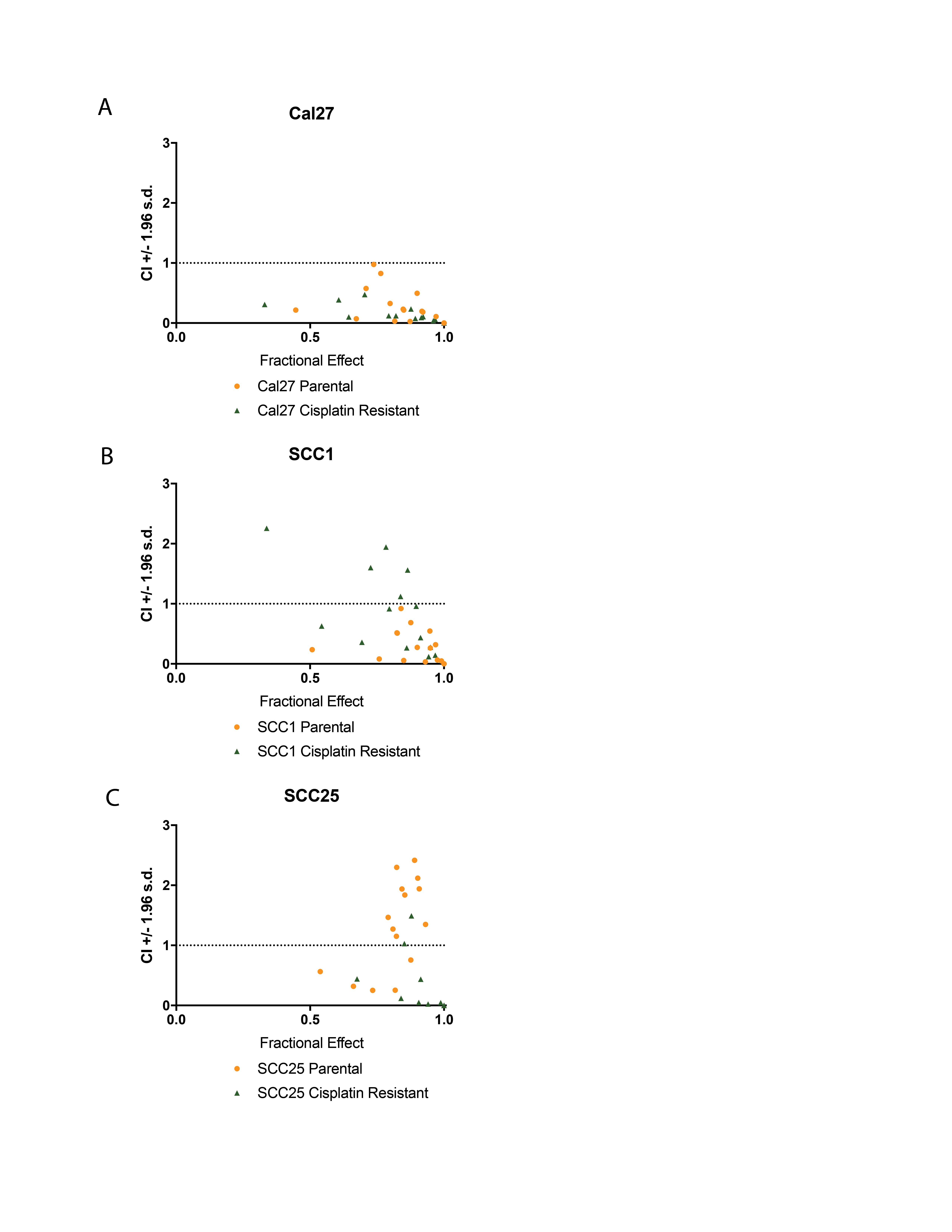

Supplement: Supplementary file 7 — Supplemental Figure 7 [file 41419_2019_2098_MOESM7_ESM.png]
