# Supplementary material for: Cisplatin exposure causes c-Myc-dependent resistance to CDK4/6 inhibition in HPV-negative head and neck squamous cell carcinoma
Source: Cell Death Dis. 2019 Nov 14;10(11):867. doi: 10.1038/s41419-019-2098-8 (PMC6856201; doi:10.1038/s41419-019-2098-8)
Supplement: Supplementary file 9 — Supplemental Legends [file 41419_2019_2098_MOESM9_ESM.docx]

**Supplemental Figure 1. In vitro sensitivity of cisplatin sensitive and resistant lines to both Cisplatin and Palbociclib.** (A) Isobologram of cisplatin after 72 hours of treatment in parental and cisplatin-resistant cell lines for Cal27, SCC1, and SCC25 (n=3). (B) Isobologram of palbociclib after 168 hours of treatment in parental and cisplatin-resistant cell lines for Cal27, SCC1, and SCC25 (n=3). Cell viability was measured by Alamar blue assay, statistics were conducted by unpaired t-test.

**Supplemental Figure 2. Palbociclib treatment causes cell cycle arrest at G1.** Cell cycle analysis for (A) Cal27, (B) SCC1, and (C) SCC25 parental and cisplatin-resistant cell lines treated with control or palbociclib for 24 hours. Cell cycle analysis for (D) CAL27, (E) SCC1, and (F) SCC25 parental and cisplatin-resistant cell lines treated with control or palbociclib for 24 hours split by cell cycle stage. (*p≤0.05, **p≤0.01, ***p≤0.001, ****p≤0.0001, by Student’s *t*-test).

**Supplemental Figure 3. Cisplatin resistant lines have heighted DNA repair capability.** (A) Quantification of comet assay in parental and cisplatin-resistant cell lines 1 hour and 4 hours after ionizing radiation (n=3, *p≤0.05, **p≤0.01, ***p≤0.001, ****p≤0.0001, by Student’s *t*-test). (B) Representative images of comet assay for Cal27 parental and cisplatin-resistant cell lines 1 hour and 4 hours after ionizing radiation.

**Supplemental Figure 4. Angiogenesis by CD31 immunohistochemical staining is decreased with palbociclib and/or JQ1 treatment.** Qualitative representative immunohistochemical staining of CD31 in tumor sections collected after 28 days of treatment with (A) vehicle control, (B) palbociclib, (C) JQ1, or (D) combination of palbociclib and JQ1. Images are representative from 2 out of 5 animals from each condition, demonstrating similar phenotype.

**Supplemental Figure 5. Expression levels of Cyclin A2 and Cyclin D1 are variable in cisplatin resistant samples, while Cyclin D1 becomes elevated with palbociclib treatment.** (A) Representative immunoblots of Cyclin A2 and Cyclin D1 for tumor cell lysates obtained from untreated cisplatin sensitive (grey) and cisplatin-resistant xenografts (black). (B) Quantification by photo densitometry was performed on bands using Image Lab Software and normalized to β-actin loading control for Cyclin A2 and Cyclin D1. (C) Representative immunoblots of cisplatin-resistant tumor cell lysates harvested after 14 days of treatment with either vehicle control (black) or palbociclib (blue) and (D) accompanying quantification by photo densitometry. (E) Tumor growth curves of cisplatin-resistant cell lines with 14 days of vehicle or palbociclib treatment. (n=5 mice per group; *p≤0.05, **p≤0.01, ***p≤0.001, ****p≤0.0001, by Student’s *t*-test).

**Supplemental Figure 6. Increased expression of c-Myc is an early event in cisplatin treatment.** (A) Representative capillary immunoblots of c-Myc for cell lysates obtained from cells treated for 1, 3, 5, 7, and 14 days with cisplatin, as well as untreated and cisplatin-resistant cell lysates. (B) Quantification by area under the curves was performed on bands using WES automated blotting system, and normalized to total protein for c-Myc.

**Supplemental Figure 7. Synergistic effects of combination of JQ1 and Palbociclib in parental and cisplatin-resistant cell lines.** Combination Index (CI) plots for interaction between JQ1 and Palbociclib administered in (A) Cal27, (B) SCC1, and (C) SCC25 parental and cisplatin-resistant cell lines. Data points on plot represent varying doses of each compound, with fractional effect calculated as percent cell death, and CI calculated using CalcuSyn software. CI>1, antagonistic effect; CI 0-1, synergistic effect.

**Supplemental Table 1. Defined values of doses of JQ1 and Palbociclib in parental and cisplatin-resistant cell lines.** Dose levels (uM) of JQ1 and Palbociclib, with corresponding fractional effect and combination index. Fractional effect calculated as percent cell death, and CI calculated using CalcuSyn software.
